# Persistent Diet-Induced Obesity in Male C57BL/6 Mice Resulting from Temporary Obesigenic Diets

**DOI:** 10.1371/journal.pone.0005370

**Published:** 2009-04-29

**Authors:** Juen Guo, William Jou, Oksana Gavrilova, Kevin D. Hall

**Affiliations:** 1 Laboratory of Biological Modeling, National Institute of Diabetes & Digestive & Kidney Diseases, National Institutes of Health, Bethesda, Maryland, United States of America; 2 Mouse Metabolism Core Laboratory, National Institute of Diabetes & Digestive & Kidney Diseases, National Institutes of Health, Bethesda, Maryland, United States of America; Institute of Preventive Medicine, Denmark

## Abstract

**Background:**

Does diet-induced obesity persist after an obesigenic diet is removed? We investigated this question by providing male C57BL/6 mice with free access to two different obesigenic diets followed by a switch to chow to determine if obesity was reversible.

**Methodology/Principal Findings:**

Male C57BL/6 mice were randomly assigned to five weight-matched groups: 1) C group that continuously received a chow diet; 2) HF group on a 60% high fat diet; 3) EN group on the high fat diet plus liquid Ensure®; 4) HF-C group switched from high fat to chow after 7 weeks; 5) EN-C group switched from high fat plus Ensure® to chow after 7 weeks. All food intake was ad libitum. Body weight was increased after 7 weeks on both obesigenic diets (44.6±0.65, 39.8±0.63, and 28.6±0.63 g for EN, HF, and C groups, respectively) and resulted in elevated concentrations of serum insulin, glucose, and leptin and lower serum triglycerides. Development of obesity in HF and EN mice was caused by increased energy intake and a relative decrease of average energy output along with decreased ambulatory activity. After the switch to chow, the HF-C and EN-C groups lost weight but subsequently maintained a state of persistent obesity in comparison to the C group (34.8±1.2, 34.1±1.2 vs. 30.8±0.8 g respectively; P<0.05) with a 40–50% increase of body fat. All serum hormones and metabolites returned to control levels with the exception of a trend for increased leptin. The HF-C and EN-C groups had an average energy output in line with the C group and the persistent obesity was maintained despite a non-significant increase of energy intake of less than 1 kcal/d at the end of the study.

**Conclusion:**

Our results illustrate the importance of considering the history of energy imbalance in determining body weight and that a persistent elevation of body weight after removal of obesigenic diets can result from very small increases of energy intake.

## Introduction

The epidemic of obesity has profound public health implications [1]. While some individuals are genetically more susceptible to develop obesity [2], [3], environmental influences have likely played a predominant role in generating the present obesity epidemic [4], [5]. Particularly important are dietary changes resulting from increased availability of palatable, high-energy foods [4]. To combat the problem of obesity, several researchers have suggested substantial re-engineering of the obesigenic environment [1], [5]. But would obesity completely reverse if the obesigenic diet was removed and replaced by a diet that would not have produced obesity in the first place? Or does the development of obesity result in a state that limits subsequent weight loss even if the environment that generated obesity has been removed?

In rodents, there are conflicting reports about the reversibility of diet-induced obesity. While some investigators have observed persistent obesity in rats [6]–[13], others have found more or less complete obesity reversal [7], [11], [14]–[18]. The results seem to depend on the duration and magnitude of weight gain – factors that were often confounded because different degrees of obesity were created by varying the length of exposure to the high energy diet [11]. In an attempt to avoid this difficulty, we investigated the persistence of diet-induced obesity in male C57BL/6 mice using two different high energy diets to generate varying degrees of weight gain before simultaneously switching to ad libitum chow to determine whether diet-induced obesity would be reversed. For the first time in mice, we found that temporary feeding of the obesigenic diets resulted in a persistent elevation of body weight and fat when compared to mice that never received the obesigenic diets. However, most of the metabolic abnormalities that were induced by the obesigenic diets were normalized. We also investigated energy balance relationships, serum metabolite and hormone concentrations, glucose and glycerol turnover rates, as well as changes of various organ and fat pad masses.

## Methods

### Animals and diets

**Figure 1:**
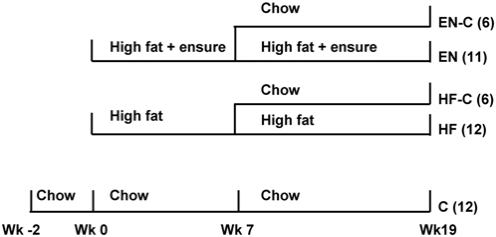
Experiment design. For the first 7 weeks two groups of weight-matched mice were fed different obesigenic diets, either a high fat solid diet or a high fat solid diet plus liquid Ensure. Subsequently, 6 mice from each group that were fed the obesigenic diets were switched to the standard chow diet for the remaining 12 weeks. All food and liquid intake was ad libitum.

Seventy one 3 month old male C57BL/6 mice weighing 25.9±1.24 g (The Jackson Laboratory, Maine) were housed individually in conditions of controlled lighting (12 hr light, 12 hr dark) and temperature (21–22°C). All mice were fed an ad libitum standard chow diet (Rodent NIH-07; Zeigler Bros., Inc. PA; 3.79 kcal/g, with 24% energy derived from protein, 12% from fat, and 64% from carbohydrate) for 14 days as an adaptation period. Six mice were euthanized for baseline measurements of organ and fat pad masses. The remaining mice were then randomly assigned to five weight-matched groups as depicted in Figure 1: 1) C group (N = 18) continued on the chow diet; 2) HF group (N = 18) on a high fat diet (F3282; Bio-Serv Inc., NJ; 5.45 kcal/g with 14% energy derived from protein, 59% from fat, and 27% from carbohydrate); 3) EN group (N = 17) on the high fat diet plus liquid Ensure® (Abbott Laboratories, Kent, UK), which had an energy density of 1.06 kcal/ml with 14% of energy derived from protein, 22% from fat, and 64% from carbohydrate; 4) HF-C group (N = 6) switched from high fat to chow after 7 weeks; 5) EN-C group (N = 6) switched from high fat plus Ensure® to chow after 7 weeks. At 7 weeks, 6 mice from the C, HF, and EN groups were euthanized to determine organ and fat pad masses and the remaining animals progressed through week 19 when all animals were euthanized. All animals received free access to water and food throughout the study. The high fat diet was provided using Rodent CAFÉ™ feeders (OYC International, Inc., MA), and liquid Ensure was provided in a 30-ml bottle with a rodent sip tube (Unifab Co., MI) and liquid intake was measured every day. Solid food intake was corrected for any visible spillage and was measured every day for the high fat diet and every other day for the chow diet using a balance with a precision of 0.01 g (Ohaus model SP402).

We certify that all applicable institutional and governmental regulations concerning the ethical use of animals were followed during this research. All procedures were approved by the National Institute of Diabetes and Digestive and Kidney Diseases Animal Care and Use Committee.

### Measurement of body composition and organ masses

Body composition was measured once per week using 1H NMR spectroscopy (EchoMRI 3-in-1, Echo Medical Systems LTD, Houston, TX) after body weight was determined. At 0, 7, and 19 weeks, 6 mice from each group were randomly selected and euthanized by cervical dislocation following i.p. administration of Ketamine/xylazine (0.1 mg/g BW of Ketamine mixed with 0.01 mg/g BW of xylazine) between 9am and 11am. Trunk blood was collected before naso-anal lengths were measured to the nearest 1 mm. Heart, kidney, spleen, liver and brown adipose tissue were harvested along with inguinal, epididymal, mesenteric, and retroperitoneal fat pads. All organs and fat pad masses were determined to within 0.1 mg precision.

### Serum metabolites and hormones

At weeks 0, 7, and 19, trunk blood from the sacrificed animals was used for the analysis of serum metabolites and hormones in the fed state. Serum insulin and leptin were measured using radioimmunoassay kits (SRI-13K and ML-82K respectively; Linco Research, St. Charles, MO). Serum glucose was measured using a Glucometer Elite (Bayer, Elkhart, IN). Serum free fatty acids, total triglycerides, and free glycerol were measured using commercially available kits from Roche Diagnostics Corp., Thermo Electro Inc., and Randox Lab Ltd., respectively.

### Ambulatory activity

Ambulatory activity was measured using the Oxymax system (Columbus Instruments, Columbus, Ohio, USA; 2.5 L chambers with wire mesh floors) with 1 mouse per chamber. The chamber sides were equipped with a mouse sensor (Opto-Varimex mini, Columbus Instruments; 16 beams of infrared at 0.5 inches apart). Ambulatory activity was recorded by counting the first beam interruption, but ignoring further interruptions unless other beams were restored or interrupted in the same time period. At the end of weeks 7 and 19, six mice from each C, HF, and EN group were housed in the chambers with free access to food and water for two days. We report the average of the measurements over the two days for each group.

### Glucose and glycerol turnover rates

Glucose and glycerol turnover were measured in restrained, conscious mice in the non-fasted state. At the beginning of the study, 6 mice were used for baseline measurements. Another 18 mice were randomly assigned to three groups on the chow, high fat diet, and high fat diet plus Ensure, respectively. At the end of study (week 19), 6 mice from each of the C, HF, and EN groups had catheters surgically inserted into their jugular veins. The procedures for catheter insertion and turnover measurements were adapted from Chen et al [19]. Two HF mice and one EN mouse did not survive the surgery. After 4–5 days of recuperation from the surgery, glucose and glycerol turnover rates were determined by continuously infusing [1–14C] glucose (3 µCi bolus, then 0.02 µCi/min) and [2–3H] glycerol (10 µCi bolus, then 0.1 µCi/min). Blood samples (30 µl) were taken from the tail vein at 0, 75, 90, 105 and 120 min. The concentrations of glucose in plasma were analyzed with a glucose oxidase method (YSI 2700 Select; Yellow Springs Instruments, Yellow Springs, OH); free glycerol was measured calorimetrically (Randox Lab Ltd, Oceanside, CA ). Glucose and glycerol concentrations in the samples between 75 and 120 min of tracer infusion were stable suggesting that a steady state was achieved. The blood samples were deproteinized with ZnSO4 and Ba(OH)2, dried to remove 3H2O, re-suspended in water, and counted in scintillation fluid (BioSafe II, Research Product International Corp., Mount Prospect, IL) on dual channels to provide separation of 3H and 14C counts for the determination of plasma [2–3H]glycerol and [1–14C]glucose, respectively. The appearance rates for glucose and glycerol were calculated using the following equation:
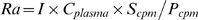
where *Ra* = appearance rate in µmol/min, *I* = infusion rate in ml/min, *S_cpm_* = scintillation counts in stock solution, *P_cpm_* = scintillation counts in plasma, and *C_plasma_* = plasma glucose or glycerol concentration in µmol/ml.

### Calculations and statistical analysis

To calculate body energy changes, ΔBE, we assumed that changes of body fat had an energy density of 9.4 kcal/g whereas fat-free mass changes had an energy density of 1.8 kcal/g [20]. Since energy is conserved, the average total energy output rate over a given time period, *T*, was calculated as:
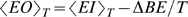
where <*EI*>*_T_* was the average energy intake over the time period, *T*. This equation is simply the energy balance equation averaged over the time interval *T*. We distinguish energy output from energy expenditure since we did not measure the energy content of feces and urine which contribute a small amount to the energy output [21]. In other words, energy output is defined as the sum of energy expenditure plus energy excreted.

Data were analyzed by analysis of variance using PROC MIXED (SAS, 1999). Tukey's method was used to adjust pairwise differences for multiple comparisons. Regression analysis was performed using the least square method. Significance was declared at P<0.05, unless otherwise noted. Means±SEM are reported and significant differences were indicated in the figures using different superscripts. The letter superscripts, a, b, c, indicate differences across the groups within each time period. The numbered superscripts, 1, 2, 3 indicate within group differences across the time periods.

## Results

### Food intake

**Figure 2:**
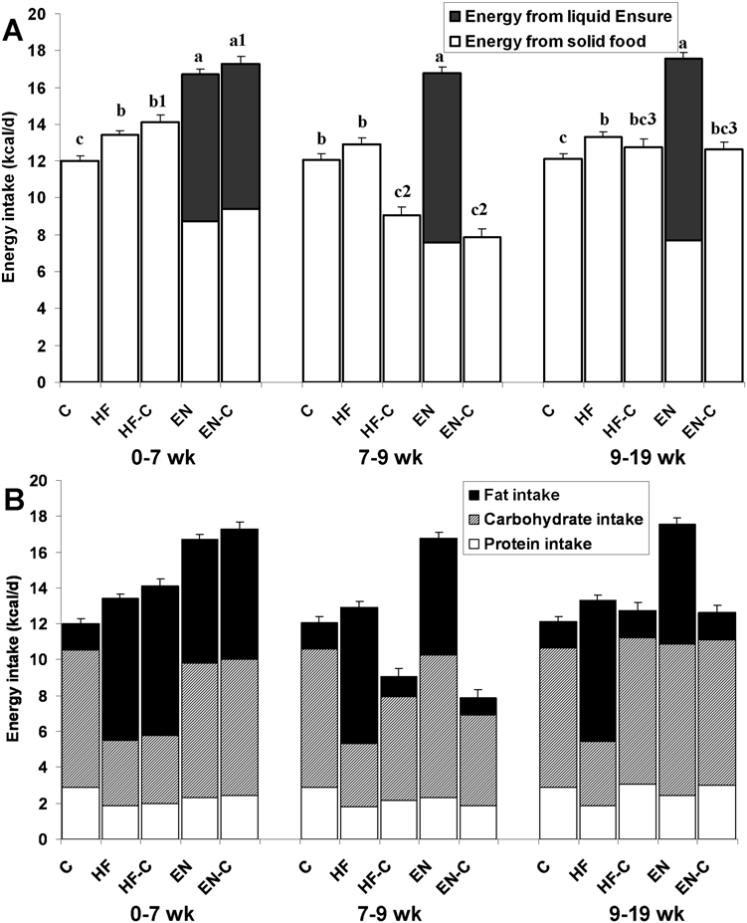
Ad libitum energy intake of male C57BL/6 mice from each diet group. The 5 diet groups: chow diet (C), high fat diet (HF), high fat diet plus Ensure® (EN), high fat diet for 7 wk followed by a switch to chow (HF-C), and high fat diet plus Ensure® for 7 wk followed by a switch to chow (EN-C). Panel A shows the energy intake derived from solid food (open bars) versus liquid Ensure (filled bars). Panel B shows the macronutrient content of the diets indicating the protein intake as open bars, carbohydrate intake as striped bars and fat intake as solid bars. Values are mean±SEM. Statistical differences among the means were determined by performing an analysis of variance. Pairwise differences were adjusted for multiple comparisons by Tukey's method. Different letters indicate significant differences of total energy intake rate across groups within each time period (P<0.05). Different numbers indicate significant within-group differences of total energy intake rate across the time periods (P<0.05).

During the first 7 weeks, the animals that were simultaneously provided with the high fat diet and liquid Ensure (the EN and EN-C groups) consumed significantly more energy than the mice given the high fat diet alone (the HF and HF-C groups; P<0.01), and all of these groups consumed more than the mice provided with the standard chow diet (the C group) (Figure 2A; P<0.01). The EN and EN-C groups had reduced solid food intake versus the HF and HF-C groups and the liquid Ensure provided about half of their energy intake. Figure 2B shows that the EN and EN-C groups consumed a similar amount of carbohydrate as the animals on the chow diet (7.46±0.13 and 7.59±0.17 vs. 7.68±0.17 kcal/day, respectively; P>0.499) and slightly less fat than the animals on the high fat diet alone (6.89±0.12 and 7.28±0.16 vs. 7.91±0.16 and 8.32±0.22 kcal/day, respectively; P<0.05).

After the switch to standard chow diet at 7 weeks, the energy intakes of the HF-C and EN-C groups fell immediately to a level significantly lower than the C group and the mice ate less than the C group over the next 2 weeks (P<0.01). During the final 10 weeks of the study, the energy intakes of the HF-C and EN-C groups were within 1 kcal/d of the C group (12.9±0.5, 12.8±0.5, and 12.1±0.3 kcal/d, respectively; P>0.15). The HF group consumed 13.3±0.3 kcal/d which was significantly higher than the C group (P = 0.01). Finally, the EN group consumed 17.2±0.4 kcal/d which was significantly greater than all other groups (P<0.01).

### Body weight

**Figure 3:**
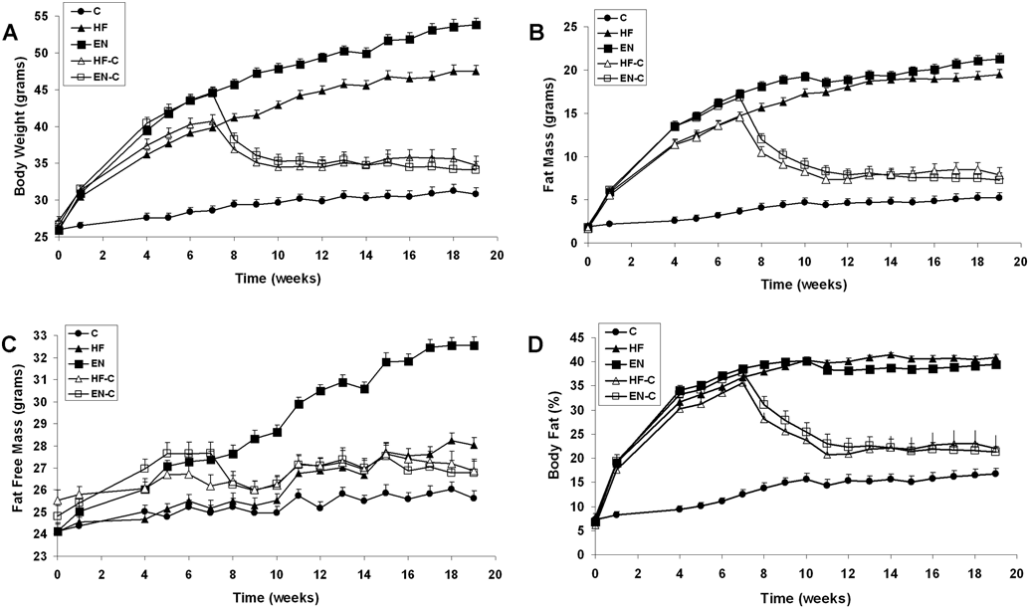
Body weight and body composition in male C57BL/6 mice from each group. Body weight (Panel A), fat mass (Panel B), fat free mass (Panel C), and percent body fat (Panel D) in male C57BL/6 mice from 5 diet groups: chow diet (C), high fat diet (HF), high fat diet plus Ensure® (EN), high fat diet for 7 wk followed by a switch to chow (HF-C), and high fat diet plus Ensure® for 7 wk followed by a switch to chow (EN-C). Values are mean±SEM and the error bars were often smaller than the size of the data point marker.

The mean body weight for each group is shown in Figure 3A. Body weight was significantly higher after 1 week of the HF and EN diets when compared with the C group (30.4±0.40, 30.9±0.41 vs. 26.5±0.40 g respectively; P<0.01). After receiving the high fat diet plus liquid Ensure® for 4 weeks, the EN group became significantly heavier than the HF group (39.5±0.58 vs. 36.2±0.58 g; P<0.01). Because the same diet was given to the HF-C and HF groups in the first 7 weeks of the experiment, these two groups had similar body weights (P>0.20). Similarly, the EN-C and EN groups had similar body weights after 7 weeks (P>0.36) which were significantly higher than the HF and HF-C groups (P<0.01).

After the switch to chow at 7 weeks, both the HF-C and EN-C groups rapidly lost weight. However, their body weight stabilized at a level significantly higher than the C group (P<0.05). Interestingly, although the HF-C and EN-C groups had different body weights at the time of diet switch, they maintained the same level of persistent obesity for the remainder of the study (34.8±1.21, 34.1±1.21 vs. 30.8±0.85 g respectively; P<0.05). At week 19, the EN group was significantly heavier than the HF group (53.8±0.89 vs. 47.5±0.85 g; P<0.01), and both the EN and HF groups had greater body weights compared with their corresponding diet-switch groups, the EN-C and HF-C groups respectively (P<0.05).

### Body fat

Figure 3B shows the mean body fat mass for each group which followed a similar pattern to the body weight curves. The EN group gained more fat mass than the HF group at week 7 (17.2±0.47 vs. 14.7±0.45 g respectively; P<0.01) as well as at week 19 (21.3±0.62 vs. 19.4±0.60 g respectively; P<0.05). The fat masses of the HF-C and EN-C groups decreased after the switch to chow, but stabilized at the same level that was significantly higher than that in the C group and remained at that level throughout the remainder of the study (7.9±0.85 and 7.3±0.85 vs. 5.2±0.60 g at week 19, respectively; P<0.05).

The percent body fat in the HF and EN groups followed a similar pattern throughout the experiment, as did the HF-C and EN-C groups (Figure 3D). The HF and EN groups had greater percent body fat than the control group at week 7 (36.8±0.76% and 38.6±0.62% vs. 12.5±0.94%, respectively; P<0.01), and both the HF and EN groups stayed at a similar body fat percentage throughout the remainder of the study (40.9±0.67% vs. 39.5±0.62% at week 19, respectively; P = 0.34). The percent body fat of the HF-C and EN-C decreased after the switch to chow diet, but remained significantly higher than that of the control group (22.0±2.70% and 21.3±1.16% vs. 16.7±1.14% at week 19, respectively; P<0.01).

### Fat-free mass and body length

Figure 3C shows that the mice receiving high fat diet plus liquid Ensure® (EN and EN-C groups) had greater fat-free mass than those on the high fat diet alone (HF and HF-C groups) or on the chow diet at week 7 (26.4±0.38 vs. 24.1±0.37 and 23.8±0.37 g respectively; P<0.01). Although the difference between the HF and C groups was not significant at week 7 (P>0.47), at week 19 the HF group gained more fat-free mass than the control group (26.6±0.37 vs. 24.0±0.37 g respectively; P<0.01) but had a lower fat-free mass than the EN group (30.7±0.39 vs. 26.6±0.37 g respectively; P<0.01). The EN-C group had a significant drop in fat-free mass (P<0.05) 1 week after the switch to the chow diet whereas the HF-C group maintained their fat-free mass. Consequently the HF-C and EN-C groups maintained almost identical fat-free masses (25.2±0.53 g for both the groups at week 19), which were significantly less than the HF group (P<0.05) but only tended to be greater than the control group (P = 0.086).

Importantly, the fat-free mass results were consistent with body length measurements. At the end of the experiment the body length in the EN group was the longest among the five groups (10.3±0.14 cm; P<0.05). The HF, HF-C and EN-C groups also tended to have increased body length compared to the control group (9.8±0.12, 9.8±0.17, 9.9±0.13 vs. 9.5±0.10 cm respectively, P<0.10).

### Energy deposition, output and ambulatory activity

**Figure 4:**
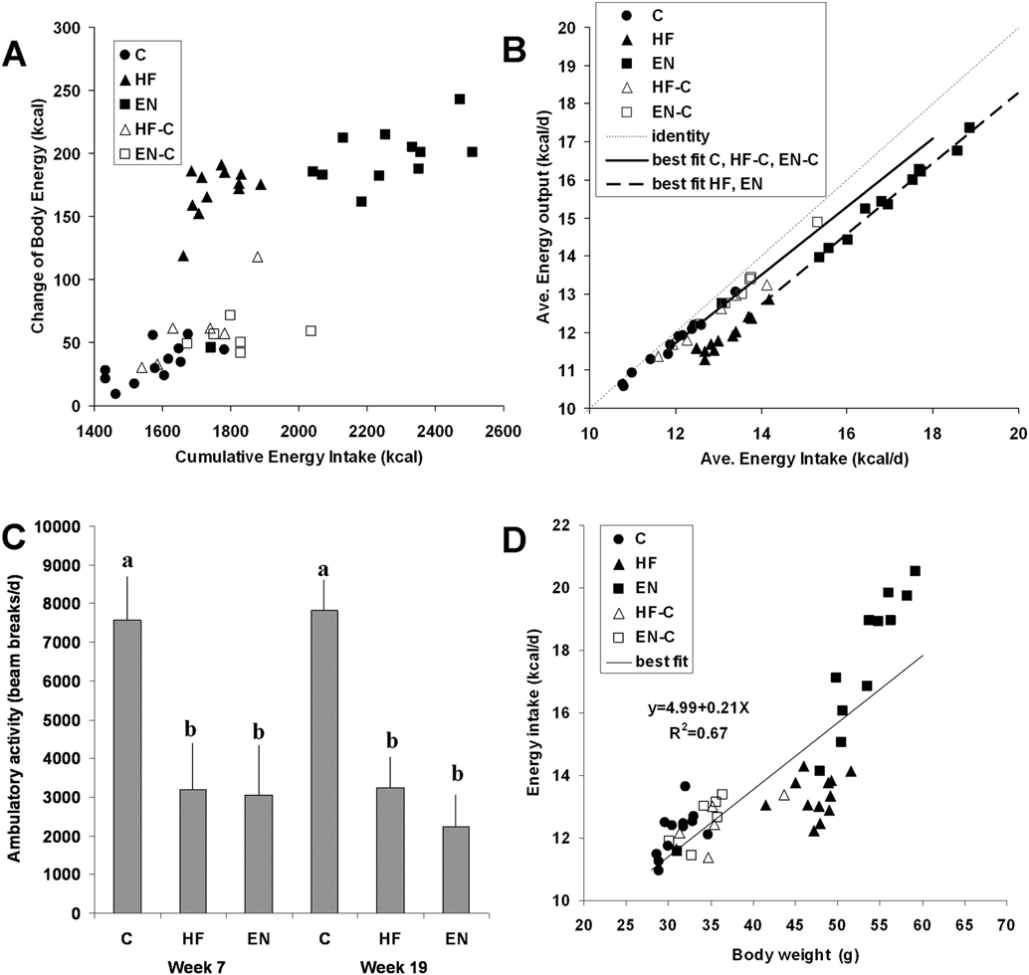
Energy intake, energy output, change of body energy, and ambulatory activity. Body energy changes versus cumulative energy intake over the 19 week experiment (Panel A) and the calculated 19 week average energy output rate versus average energy intake rate (Panel B). The data points indicate individual mice from the 5 diet groups: chow diet (C), high fat diet (HF), high fat diet plus Ensure® (EN), high fat diet for 7 wk followed by a switch to chow (HF-C), and high fat diet plus Ensure® for 7 wk followed by a switch to chow (EN-C). In panel B, the dotted line is the line of identity indicating average energy balance. The solid line is the best-fit regression line for the C, HF-C, and EN-C groups (slope = 0.90±0.03, intercept = 0.97±0.44 kcal/d, R2 = 0.98) and the dashed line is the best-fit regression line for the HF and EN groups (slope = 0.92±0.02, intercept = −0.21±0.28 kcal/d, R2 = 0.99). C) The average ambulatory activity of the mice measured at weeks 7 and 19. The bars with different letters indicate significant differences of ambulatory activity within each time period (P<0.05). Panel D plots the energy intake rate over the last two weeks of the study as a function of the final body weight (slope = 0.21±0.02 kcal/g/d, intercept = 4.99±0.93 kcal/d, R2 = 0.67). The regression analysis was performed using the least square method and other statistical analyses were performed as in Figure 2.

Figure 4A plots the gain of body energy as a function of the cumulative energy intake for the individual animals in each diet group after 19 weeks. There was clearly a positive correlation between energy intake and body energy gain (r = 0.74, P<0.01), but the body energy gain in the HF and EN groups was disproportionately higher than the other groups indicating increased energy deposition efficiency in these mice (P<0.01).

To further explore this observation, we calculated the average energy output as a function of the average energy intake as depicted in Figure 4B. The dashed line is the line of identity indicating a state of average energy balance. The data clearly separated into two lines relating average energy output and energy intake rates, with the HF and EN animals falling on a line below that of the other groups. Regression analysis revealed similar slopes for the two lines (slope = 0.92±0.02 vs. 0.90±0.03, P = 0.45), but the y-intercept was significantly decreased for the HF and EN animals (−0.21±0.28 vs. 0.97±0.44 kcal/d; P<0.01). Therefore, feeding the obesigenic diets decreased the average energy output rate by 1.2±0.5 kcal/d after adjusting for energy intake (P<0.05). The relative decrease of the average energy output rate of the HF and EN groups corresponded to significantly lower average ambulatory activity versus the C group (Figure 4C) implying that at least part of the effect of the obesigenic diets on average energy output was due to decreased ambulatory activity.

Figure 4D shows that the average energy intake rate of the mice over the last two weeks of the study was positively correlated with body weight (r = 0.82, P<0.01) and the slope of the regression line was 0.21±0.02 kcal/g/d. Since the body weight was relatively steady at the end of the study, the energy output rate was approximately equal to the energy intake rate and therefore energy output was also positively correlated to body weight according to the same relationship.

### Organ and fat pad masses

Table 1 reports the liver, spleen, kidney, and heart masses for each diet group at weeks 0, 7, and 19. After 7 weeks, the high fat plus liquid Ensure diet resulted in increased liver, spleen, and heart masses versus chow or high fat diet alone (P<0.05). The kidney mass also tended to be higher in the EN group versus the C group (P = 0.065). These organ mass differences were more pronounced by week 19 (P<0.05). High-fat feeding alone had a significant effect on the heart mass, which was 28% higher in the HF group compared to the C group at week 19 (P<0.05). In the HF-C and EN-C groups, liver, spleen, kidney, and heart masses were all normalized at week 19.

**Table 1:** Organ masses.

Period	Diet group	Liver (g)	Spleen (g)	Kidney (g)	Heart (g)
Week 0	C	1.30±0.125	0.08±0.008	0.34±0.027	0.13±0.007
Week 7	C	1.47±0.086^b^	0.08±0.005^b^	0.36±0.038	0.13±0.015^b^
	HF	1.54±0.074^b^	0.08±0.008^b^	0.41±0.062	0.15±0.008^b^
	EN	2.32±0.350^a^	0.11±0.016^a^	0.42±0.043	0.19±0.048^a^
Week 19	C	1.54±0.123^b^	0.09±0.009^b^	0.42±0.035^bc^	0.14±0.020^c^
	HF	2.30±0.352^b^	0.10±0.007^b^	0.41±0.030^c^	0.18±0.019^b^
	HF-C	1.67±0.235^b^	0.09±0.012^b^	0.47±0.021^ab^	0.15±0.008^bc^
	EN	5.23±1.592^a^	0.17±0.020^a^	0.51±0.071^a^	0.20±0.035^a^
	EN-C	1.67±0.196^b^	0.09±0.008^b^	0.45±0.058^bc^	0.15±0.012^c^

Data are presented as means±SEM. N = 6 for all the groups except for the EN group at week 19 where N = 5. Within each period, different letters indicate significant differences between treatments (P<0.05).

Table 2 shows how body fat was distributed across the major fat pads: mesenteric, retroperitoneal, epididymal, inguinal, and brown adipose tissue. As expected, all fat pads were enlarged in the HF and EN groups at weeks 7 and 19 compared with the C group. At week 7, the EN group had increased brown and mesenteric adipose tissue versus the HF group, but the mass of epididymal fat tended to be greater in the HF group versus the EN group at week 7 (P<0.10), and this difference became more pronounced by the end of the study (P<0.05). Interestingly, the epididymal fat pad mass decreased by more than 25% in both the HF and EN groups from weeks 7 to 19 (P<0.05) despite the overall increase of body fat mass.

**Table 2:** Fat pad masses.

Period	Diet group	BAT (mg)	MES (mg)	RET (mg)	EPI (mg)	ING (mg)
Week 0	C	92±18	193±43	109±30	404±75	324±62
Week 7	C	114±8^c^	229±66^c^	265±109^b^	711±201^b^	538±161^b^
	HF	265±24^b^	1009±145^b^	1091±166^a^	2848±266^a^	1760±175^a^
	EN	462±31^a^	1462±323^a^	1282±309^a^	2468±532^a^	1759±458^a^
Week 19	C	163±17^c^	352±78^d^	429±164^c^	1053±266^d^	666±145^c^
	HF	451±20^b^	1605±121^a^	1745±239^b^	2094±138^a^	2821±144^a^
	HF-C	193±80^c^	511±197^c^	629±205^c^	1404±334^bc^	1042±549^b^
	EN	743±128^a^	1304±64^b^	2444±548^a^	1681±440^b^	2729±185^a^
	EN-C	201±43^c^	391±97^cd^	428±88^c^	1134±103^cd^	949±257^bc^

Abbreviation: BAT: brown adipose tissue, MES: mesenteric fat pad, RET: retroperitoneal fat pads, EPI: epididymal fat pads, ING: inguinal fat pads. N = 6 for all the groups except for the EN group at week 19 where N = 5. Values are means±SEM. Within each period, different letters indicate significant differences between diet groups (P<0.05).

After switching to the chow diet, the HF-C and EN-C groups had decreased fat pad masses versus the HF and EN groups at week 19 (P<0.05). However, the HF-C group retained higher mesenteric, epididymal, and inguinal fat pad masses versus the C group at week 19 (P<0.05). Although not statistically significant, the EN-C group had a trend for higher inguinal fat pad mass compared with the control group (P = 0.12).

### Blood parameters

After 7 weeks, the high fat plus Ensure® diet significantly increased serum glucose, insulin, and leptin concentrations and decreased total triglyceride concentrations in the EN group versus the C group (P<0.05; Table 3). Feeding the high fat diet alone significantly increased the serum glucose and leptin levels and decreased serum triglyceride concentrations in the HF versus C groups (P<0.05). These changes were further exacerbated after 19 weeks (P<0.05). Twelve weeks after the diet switch, the HF-C and EN-C groups normalized all blood parameters with the exception of a trend for higher circulating leptin concentrations (P = 0.13 and 0.28 for HF-C and EN-C vs. C, respectively).

**Table 3:** Serum glucose, NEFA, glycerol, triglycerides, leptin, insulin concentrations.

Period	Diet group	Glucose (mg/dl)	FFA (mM)	Glycerol (µM)	Triglyceride (mg/dl)	Leptin (ng/ml)	Insulin (ng/ml)
Week 0	C	229±15	0.28±0.014	197±14	76.5±3.58	3.3±0.43	0.2±0.01
Week 7	C	229±8^b^	0.27±0.018	233±14	79.7±4.76^a^	6.9±1.10^b^	0.8±0.18^b^
	HF	283±8^a^	0.30±0.085	242±36	47.2±5.28^b^	57.8±13.0^a^	1.3±0.41^b^
	EN	300±22^a^	0.31±0.056	268±31	53.6±7.29^b^	70.2±17.9^a^	3.9±2.68^a^
Week 19	C	279±39^b^	0.26±0.059^b^	212±52^bc^	84.7±24.1^ab^	9.8±3.6^b^	0.7±0.14^b^
	HF	344±47^a^	0.37±0.068^a^	255±25^ab^	51.2±16.2^c^	82.6±15.1^a^	2.3±0.42^b^
	HF-C	278±39^b^	0.32±0.139^ab^	189±50^c^	90.3±27.6^a^	19.0±11.4^b^	0.9±0.32^b^
	EN	352±33^a^	0.39±0.081^a^	308±39^a^	65.3±18.7^bc^	84.5±11.4^a^	6.8±5.34^a^
	EN-C	268±41^b^	0.26±0.048^b^	196±23^c^	85.1±19.9^ab^	16.4±6.1^b^	1.0±0.40^b^

Values are means±SEM. Within each period, different letters indicate significant differences between diet groups (P<0.05). Week 19 glycerol C vs. EN, P = 0.1164; week 19 leptin HF vs. EN, P = 0.1245; N = 6 for all the groups except for the EN group at week 19 where N = 5.

### Glycerol and glucose turnover rates

Table 4 shows that the rates of glucose and glycerol turnover in the C group did not change from week 7 to week 19 (P>0.3). However, after 19 weeks the mice in the HF and EN groups had greater glycerol and glucose appearance rates compared to the control animals (P<0.05). No significant difference was observed between the HF and EN groups. However, there was a marginal increase of the glucose rate of appearance in the EN group compared with the HF group (P = 0.127). Regression analysis revealed that the glucose turnover rates were correlated with fat-free mass across all the groups (r = 0.85, P<0.01) and glycerol appearance rates were correlated with body fat mass (r = 0.62, P<0.01).

**Table 4:** Glycerol and glucose turnover rates.

Period	Diet group	Glycerol Appearance Rate (µmol/min per mouse)	Glucose Appearance Rate (µmol/min per mouse)
Week 0	C	0.212±0.021	2.97±0.45
Week 19	C	0.217±0.025^b^	3.06±0.40^b^
	HF	0.312±0.072^a^	4.36±0.98^a^
	EN	0.298±0.081^a^	5.04±0.50^a^

Glucose Appearance Rate HF vs. EN, P = 0.1266. N = 4, 5, 6 and 6 for the HF, EN, and the two control groups respectively. Values are means±SEM. Within each period, different letters indicate significant differences between diet groups (P<0.05).

## Discussion

This is the first report of persistent diet-induced obesity in mice. Two previous studies also using C57BL/6 mice did not observe persistent obesity following removal of high fat diets [17], [18]. But these previous studies used younger mice (4–6 weeks of age vs. 3 months old in the present study) that were housed in groups of 5 animals per cage as opposed to individual housing. These factors, along with differences in the composition of the diets, likely contributed to our unique observation that returning mice to a non-obesigenic diet after 7 weeks of exposure to two different obesigenic diets resulted in sustained elevations of body fat by 40–50% when compared with mice that were never exposed to the obesigenic diets.

Of course, the persistence of obesity for 12 weeks following the return to a non-obesigenic diet does not prove that these mice would have permanent alterations of body weight and composition. Nevertheless, this period of time represents a significant fraction of the average lifespan of these mice (about 10 percent) and was more than 70% longer than the period of obesity induction. Furthermore, unlike the previous observations of persistent diet-induced obesity in rats that occurred on a background of significant growth [6]–[13], our chow fed mice increased their fat-free mass by less than 5% over the 19 week study. Thus, beginning the study with 3 month old mice achieved relative stability of lean tissue mass in the control group and allowed diet interventions of sufficient duration while avoiding confounding factors of advanced age.

Interestingly, the different average body weights and fat masses attained after 7 weeks of HF versus EN diets did not impact the final level of persistent obesity (Figure 3). This suggests that both diets resulted in sufficient weight gain to cross a threshold for inducing a fixed increment of persistent body weight gain. In contrast, a graded effect would be expected to result in different levels of persistent obesity for varying degrees of diet-induced weight gain. This did not happen. Rather, our observations are consistent with a previously suggested mechanism of inducing multiple body weight set points generated by acquired leptin resistance during the high energy diets [22], [23]. Future studies could potentially locate the threshold weight gain required to achieve persistent obesity by varying the dietary fat percentage to scan a range of body weight gains over the 7 week obesigenic period.

To help understand the energy imbalances underlying our results, we used the principle of energy conservation to calculate the average energy output rate as a function of the average energy intake rate (Figure 4B). This analysis revealed that HF and EN mice had decreased average energy output relative to their average energy intake. Decreased average ambulatory activity in the HF and EN mice (Figure 4C) likely contributed to this relative reduction of energy output and similar behavior was recently observed in C57BL/6 mice fed with a high fat diet [21]. The mice in the diet switched groups, HF-C and EN-C, had average energy output rates that fell on the same line as the C group implying that their average energy output was comparable to the control group.

The HF-C and EN-C mice cumulatively consumed more energy than the control mice, but there were no statistically significant differences between the energy intake rates of the HF-C, EN-C, and C groups over the last several weeks of the study (Figure 2A) despite the steady elevation of body weight. While not statistically significant, the energy intake rates of the HF-C and EN-C mice at the end of the study were numerically greater than the C group, but within 1 kcal/d. Significant differences of body weight can be maintained by very small differences of energy intake thereby putting severe constraints on the ability to experimentally detect such differences. For example, the slope of the regression line relating energy intake and body weight at the end of our experiment (Figure 4D) was only 0.21±0.02 kcal/g/d which means that less than 1 kcal/d of additional energy intake would be required to maintain the 4 g of additional body weight observed in the HF-C and EN-C groups at the end of the study. While this difference of body weight is easily measured, detecting such a small difference of energy intake or output is technically challenging. Similar considerations suggest the potential importance of alterations of gut absorption [24], [25] resulting in changes of energy excretion in urine and feces which were not directly measured in the present study, but were included in our calculations of energy output.

These observations highlight an important fact that is often overlooked in studies of obesity: changes of body weight indicate past cumulative energy imbalance and understanding the genesis of body weight differences requires knowledge about this history in addition to measurements made at isolated time points [26]. Because the relationship between steady-state energy expenditure versus body weight has such a shallow slope, isolated measurements of energy intake or output rates may be unable to detect statistically significant differences between groups that have clearly different body weights. This difficulty may lead to the potentially erroneous conclusion that the lack of a statistically significant difference implies that there is in fact no difference and therefore altered energy efficiency must underlie the body weight differences – often illustrated by somehow normalizing the measurements for body weight [27]. In contrast, by taking into consideration the history of energy imbalance, the present study suggests that very small increases of energy intake likely explain the persistent obesity observed in the HF-C and EN-C mice.

The pattern of body fat accumulation in obesity is believed to play a role in disease risk [28]–[31] and we found interesting dynamics of body fat accumulation in the various fad pads during the development of obesity (Table 2). Both obesigenic diets led to dramatic increases of all fat pads at weeks 7 and 19 versus the modest increases observed in the chow group. However, not all fat pads monotonically increased in size on the obesigenic diets. Rather, the epididymal fat pads decreased by more than 25% between weeks 7 and 19 in the HF and EN groups despite increases of the other fat pad masses as well as overall body fat. This observation is consistent with a recent report showing a non-monotonic relationship between epididymal fat mass and body weight that was associated with adipose tissue remodeling during the development of diet-induced obesity [32]. In the mice that were switched from the obesigenic diets back to chow, all fat pad masses were decreased at week 19 in comparison to their corresponding masses at week 7. However, in reflection of their persistent obesity, the HF-C group retained significant elevations of mesenteric, epididymal, and inguinal fat pad masses versus the C group at week 19 and a similar trend was observed in the EN-C group.

Both obesigenic diets resulted in increased serum leptin, insulin, and glucose, but triglycerides were paradoxically lower. After the switch to chow, all serum measurements returned to control values at week 19, with the exception of leptin which tended to remain increased in the persistent obese HF-C and EN-C mice versus the control group (Table 3). The HF and EN mice that continued their obesigenic diets for the entire 19 weeks had further deterioration of their metabolic state with increased serum glucose, insulin, and leptin concentrations while curiously maintaining lower serum triglycerides (Table 3).

Paradoxically decreased circulating triglycerides (TG) have been previously found in high fat fed C57BL/6 mice despite their insulin resistant state [33]. While reduced dietary carbohydrate might explain these previous observations and why we also found lower serum TG in the HF group versus the C group, the EN group consumed the same amount of carbohydrate as the C group but also had decreased serum TG. Since both the EN and HF groups consumed similarly high amounts of dietary fat, perhaps the increased fat intake (rather than the reduced carbohydrate intake) caused the observed lowering of circulating triglycerides either by suppressing TG production and/or increasing TG clearance. Further support for this hypothesis comes from the study by Meugnier et al. who measured decreased circulating TG concentrations after adding 550 kcal/d of fat to the daily diet of lean men while keeping carbohydrate intake constant [34].

A possible alternative mechanism for the reduced serum TG levels could have been the elevated insulin concentrations observed in both the HF and EN groups since insulin is known to acutely inhibit hepatic VLDL secretion [35] and stimulate adipose TG uptake via LPL [36]. However, chronic elevations of insulin consequent to insulin resistance typically go along with high VLDL secretion and correspondingly high circulating TG [37]. Furthermore, both HF and EN groups had increased rates of whole body lipolysis as reflected by elevated glycerol appearance rates (Table 4) and increased serum glycerol and FFA concentrations (Table 3). Thus, adipose tissue was not responding to the elevated insulin and the resulting increased FFA supply to the liver would be expected to increase VLDL secretion and thereby increase rather than decrease serum TG. Clearly, more work is required to understand the mechanism for lowering serum TG during high fat feeding.

The cause of the obesity epidemic is typically attributed to an environment that promotes excessive food intake and limits physical activity [1], [5]. Thus, a possible solution to the obesity problem involves re-engineering the obesigenic environment towards one that would not have resulted in obesity. The present study raises the possibility that even such a dramatic change of the environment may not be sufficient to completely reverse obesity. Nevertheless, the return to the non-obesigenic environment allowed the persistently obese mice to normalize their previously increased serum concentrations of glucose, insulin, leptin, free fatty acids, and glycerol, while their paradoxically low serum triglycerides increased to normal values. While the energy intake rate of the persistently obese mice was within 1 kcal/d of the control mice at the end of the study, we believe that such a small increase likely explains the persistent elevation of body weight and fat mass.
